# Vitamin D Status and Associated Factors in Neonates in a Resource Constrained Setting

**DOI:** 10.1155/2018/9614975

**Published:** 2018-07-05

**Authors:** Khadija Murtaza Bhimji, Helga Naburi, Said Aboud, Karim Manji

**Affiliations:** ^1^Departments of Paediatrics and Child Health, Muhimbili University of Health and Allied Sciences, Tanzania; ^2^Microbiology and Immunology, Muhimbili University of Health and Allied Sciences. P.O. Box 65001, Dar es Salaam, Tanzania

## Abstract

Vitamin D deficiency (VDD) is emerging as a serious public health problem globally; however due to lack of resources, vitamin D levels are not routinely measured among neonates. The study was conducted to determine vitamin D levels in neonates and factors associated with the same. A cross-sectional study was conducted among neonates admitted at neonatal ward of a tertiary care hospital. Means and proportions were calculated from summarized data in frequency tables. Chi square test was used to determine association between vitamin D and various associated factors such as sex, infant birth weight, gestation age, parity of the mother, maternal age, and HIV status of the mother. A total of 170 neonates were studied, out of which 80% had vitamin D deficiency. Neonates born to HIV-infected mothers were significantly less likely to have vitamin D deficiency (OR 0.21, 95% CI 0.06 – 0.77, p = 0.009). Subgroup analysis revealed the association to be stronger in terms neonates (p = 0.005). The association was not observed among preterm newborns. The prevalence of vitamin D deficiency in neonates was observed to be very high and needs more attention.

## 1. Introduction

Vitamin D deficiency (VDD) is a worldwide problem that has been documented among neonates, ranging from 73% in New Zealand [1] to 94% of newborns in Jordan [2].

Some of the risk factors associated with VDD such as HIV infection, teenage pregnancy, preterm, and low birth weight deliveries are prevalent in our setting [3, 4].

VDD has multiple consequences and is increasingly recognized as a factor associated with various diseases. In children, it leads to growth retardation and rickets. Nonskeletal consequences of VDD include an increased risk of certain cancers, hypertension, type 1 diabetes mellitus, multiple sclerosis, and more severe forms of tuberculosis [5].

In Africa, VDD and related consequences are not uncommon; for instance, in Kenya, incidence of rickets of prematurity by six months of age was found to be 58.8% and was more common in male infants compared to female infants [6].

Similarly in Tanzania, a study done by Msomekela et al. concluded that metabolic bone disease is common in very low birth weight, exclusively breastfed babies with almost 33% of the study population having clinical rickets at 12 weeks [7]. This study was conducted to find out the vitamin D status among neonates of different birth weights and gestational age and to identify neonatal and maternal factors associated with it.

## 2. Materials and Methods

This hospital based cross-sectional study was carried out between April and June 2015 among neonates admitted at the Neonatal Ward at Muhimbili National Hospital (MNH). MNH is one of the four tertiary hospitals in the country. It is also a tertiary level referral for three municipal hospitals in Dar es Salaam and serves as a University teaching hospital for Muhimbili University of Health and Allied Sciences (MUHAS). The neonatal ward has a bed capacity of 120 beds with 20-25 admissions a day.

The standard definitions for parity, birth weight, and gestational age were used.

Inclusion criteria for study participants were neonates admitted at MNH, from whose parents informed consent to test was obtained within 24 hours. Neonates with known sepsis, renal disease, liver disease, jaundice, and/or on phototherapy were excluded from the study, as they can affect the vitamin D status.

All neonates that met the inclusion criteria were consecutively included in the study population.

### 2.1. Data Collection

On admission, each participant had details of age, gestation, birth weight, and relevant information like breastfeeding, collected in a structured questionnaire. Likewise, the mothers also had their sociodemographic details including HIV status documented. Before each weight measurement, the weighing scale was calibrated to zero and the neonate was weighed without any clothing.

### 2.2. Laboratory Investigation

Blood samples for vitamin D testing were taken within 24 hours of admission and were transported to laboratory within 2 hours of collection. Blood samples were aliquoted at 1500 rev/minute for 15 minutes and sera were then aliquoted and stored in -86°C freezer until the time for assay. Vitamin D levels were measured using 25(OH) EIA (Immunodiagnostics, UK). Briefly, serum sample was dispensed in wells in duplicate and Biotin reagent was added. The sample was incubated for 2 hours. The microtitration wells were washed three times using the Wellwash 4MK2 system. Following 30 minutes of incubation, an enzyme conjugate was added that was followed by another 30 minute incubation. The wells were then washed thrice and tetra-methylbenzidine was added. Diluted hydrochloric acid (HCL) was added after 30 minutes and the microtitration plate was loaded in the Multiscan Ex ELISA reader. Serum vitamin D levels were determined by measuring the optical density of the individual wells. Calibration curve was prepared and vitamin D levels were calculated and expressed in nmol/L.

In every run, seven calibrators (calibration 0-6) and two levels of controls (normal and high) were included and run in duplicate. Assay run was considered valid if all 7 calibrators and controls were within the acceptable range. Testing was performed in the MUHAS Clinical Research Laboratory, Department of Microbiology and Immunology at MUHAS. The laboratory participates in the proficiency testing program called DEQAS to ensure accuracy and reliability of results.

The testing laboratory technicians were blinded to the characteristics of each participant to avoid bias of results. The following definitions were used for the vitamin D levels: deficiency: vitamin D levels ≤49 nmol/L, insufficiency levels between ≥50 and ≤74 nmol/L, and vitamin D sufficient levels ≥75 nmol/L [8].

### 2.3. Ethical Considerations

Ethical clearance and permission to conduct the study were obtained from MUHAS Senate Research and Publications Committee and MNH administration, respectively (Ref No. MU/PGS/SAEC/Vol.XIV, www.muhas.ac.tz).

Parents/caretakers of the study participants were requested to sign informed consent forms prior to the enrolment into the study.

All the neonates received treatment as per the Ministry of Health Community Development, Gender, Elderly and Children (MOHCDGEC) guidelines regardless of their choice of participating in the study.

The results were shared with the clinicians in the newborn unit and those who were found to be vitamin D deficient were supplemented with cod liver oil 2.5 millilitres (equivalent to 400 I.U) once a day for 12 weeks. The infants are scheduled for a monthly infant clinic visit and the clinician will continue managing the infant including decision to stop vitamin D supplementation.

### 2.4. Data Analysis

Data were analyzed using Stata program version 12.0. Chi square and Fisher's exact test where applicable were used to determine the association between VDD and the various risk factors, namely, parity of the mother, maternal age and HIV status of the mother, infant sex, birth weight, gestation age, and existing comorbidity. Odds ratio was used to determine the association between the associated factors and VDD. Subgroup analysis was performed to determine the effect of factors associated with preterm and term neonates, separately. A p-value <0.05 was regarded as statistically significant. For the purpose of analysis, neonates with vitamin D sufficiency and insufficiency were grouped as one category (vitamin D sufficient) because of the very few number of neonates with sufficient vitamin D levels (3 out of 170 neonates).

## 3. Results

Of the 170 neonates that were studied, 106 (62.3%) were term neonates and 62 (36.5%) were premature. There were slightly more male neonates (51.8%) than females. Neonates born with birth weight of more than 2500gm accounted for 75.3% of the study population. Half of the neonates received breast milk with colostrum (50.0%). (Table 1)

Out of the normal term newborns, 75 (44.1%) had no known comorbidities. The remaining newborns were admitted due to various reasons including birth asphyxia (11.2%), transient tachypnoea of the newborn (14.8%), and others.

**Figure 1:**
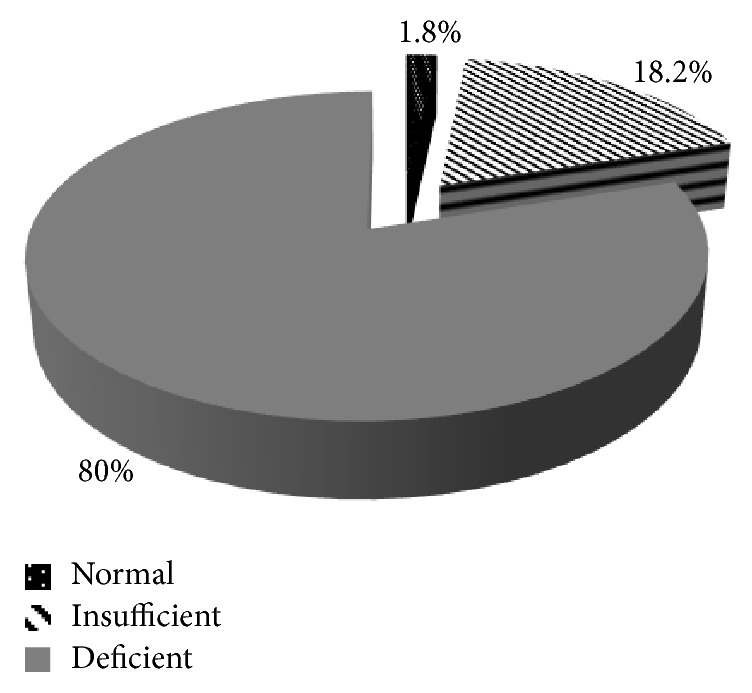
Vitamin D levels in the study population.

Of the 170 neonates that were included in the study, 3 (1.8%) had normal vitamin D levels, 31 (18.2%) had insufficient vitamin D levels, and 136 (80.0%) had vitamin D deficiency (see Figure 1).

Among neonates born to HIV-infected mothers, 50% had VDD, compared to 82.7% of neonates with VDD born to HIV uninfected mothers. The difference was statistically significant (p = 0.009) (Table 2).

Female neonates had a slightly higher proportion of VDD (85.4%) compared to males (75.0%). Neonates born premature had lower proportion of VDD (77.4%) compared to term neonates (81.5%). Among neonates with birth weight less than 2500gm, 81.0% had VDD compared to 79.7% of neonates with birth weight of more than 2500gm. Of 62 neonates born to primiparous mothers, 82.3% had VDD compared to 78.8% and 77.8% of neonates born to multiparous and grand multiparous mothers, respectively. Neonates born to mothers less than 20 years of age, 87.5% had VDD compared to 80.9% and 76.7% of neonates with VDD born to mothers of 20-29 years of age and those with 30 years of age and more, respectively. Among neonates who were breastfed with colostrum, 77.6% had VDD compared to 100.0% of neonates with VDD who were breastfed without colostrum and 80.5% of neonates with VDD who were not breastfed at all. However, all these differences were not statistically significant (p > 0.05) (Table 2).

Table 3 shows neonatal vitamin D status according to HIV status of the mother. Neonates born to mothers who were HIV-infected were 5 times less likely to have VDD as compared to neonates born to mothers who were HIV uninfected and this was statistically significant (p = 0.009).

VDD among preterm born to HIV-infected mothers was 66.7% compared to 78.6% among HIV uninfected mothers (p = 0.61) (Table 3). Term neonates born to mothers who were HIV-infected were 10 times less likely to have VDD as compared to neonates born to mothers who were HIV uninfected (OR = 0.11) and this was statistically significant (p = 0.005) (Table 3).

Infants who had an existing all-cause morbidity had higher prevalence of VDD (81%) compared to 78.7% among those without comorbidity (p=0.61) (Table 4).

## 4. Discussion

This was a cross-sectional study aimed at identifying VDD and its associated factors in neonates at a tertiary care hospital in Tanzania.

VDD was found to be a big burden in this population, present in 80% of the neonates studied, despite more than half being breastfed and majority being given colostrum. Only 3 neonates had sufficient vitamin D levels. This high burden of VDD among neonates has also been found in previous studies conducted in Tanzania [9], Jordan [2], Pakistan [10], and Northern India [8]. These findings emphasize that VDD among neonates is a significant global problem that requires immediate attention in order to prevent the complications that have also been reported in these resource constrained countries [6, 7].

There are some possible reasons to explain the high burden of VDD in these neonates. First, several studies have shown a positive correlation between maternal and newborn vitamin D levels [11–14].

The possible reason for the mothers to have low vitamin D levels could be because all mothers in this study were dark-skinned. This association between VDD and dark-skinned individuals was also reported in USA [15]. Another reason could be that the population of mothers in this study were from suburban areas where women are normally indoors and travel in buses, which reduces their exposure to sunlight.

These findings suggest that there may be a role for maternal supplementation of vitamin D during pregnancy in order to reduce the burden of VDD in the neonate [16–19].

Interestingly, in this study, HIV positive status was not associated with VDD, rather it seemed protective. This is in sharp contrast with previous studies conducted by Mehta et al. [20] and Sudfeld et al. [9] in HIV positive cohorts. This could be possibly because of the difference in the sample size and selection bias. It is possible that HIV-infected mothers who had a good health seeking attitude attended the antenatal clinics and thus had better chances of good health. Moreover, studies done by the previous researchers were done during the era when antiretroviral drugs (ARVs) were not being actively provided to pregnant women. So, our HIV-infected mothers may have had a relatively good health, as patients in the HIV program, including mothers, are also provided with vitamin supplements [21]. Furthermore, good health of HIV-infected mothers due to counselling, provision of multivitamins at care and treatment clinics, and more awareness of their status could have contributed to these contrasting findings [21]. Therefore, this requires further deliberation.

It may also be possible that sufficient vitamin D levels in HIV exposed infants may be due to an acute phase reaction in the neonate [9]. The number of neonates in this study with these findings are small and need further large-scale studies and analysis.

Other factors that were assessed in the study included sex of the neonate, gestational age (GA), birth weight, breastfeeding status, maternal age, and parity. None of these factors were seen to be associated with VDD in the neonate. These findings are in line with studies done in Turkey [22], Greece [23], and Brazil [24], where GA, birth weight, sex of the neonate, maternal age, and parity were not shown to be associated with VDD in the newborn. However, in Jordan, lower GA was found to be associated with VDD in the neonate [2]. This difference could be due to the smaller sample size in this study in contrast the large sample size used in the study done in Jordan.

Although there was no significant association between VDD and prematurity, the fact that prematures already show VDD and that these are high risk of developing metabolic bone disease [7], it is therefore a need to consider supplementation as soon as possible and as a matter of policy. In neonatal units in the USA, neonates are supplemented with 400 IU of vitamin D during the first year of life, as per as the recommendations from the American Academy of Paediatrics [11].

In our neonatal unit, premature and HIV exposed newborns are supplemented with a multivitamin formula that consists of 400 IU of vitamin D (in each 0.6mls), other essential vitamins, and Omega 3 from fish oil. This is based on recommendations from the national guidelines [25].

It is interesting to note that from all factors studied, none of the neonatal factors had any association with the vitamin D status in the neonate. Maternal HIV had an association on vitamin D status. This further emphasizes the role of antenatal supplementation of mothers in order to prevent VDD in the neonates [16–19]).

The strength of this study is that it is the first of its kind in Tanzania and gives a picture of the burden of VDD in the local setting. It also studied the association of various potential factors that can influence vitamin D levels in newborns. This can help in planning and allocation of resources and future clinical trials. All neonates who were found to have low levels of vitamin D in this study were treated appropriately.

There were some limitations in this study. Maternal vitamin D levels were not measured and hence it is unclear whether maternal vitamin D levels accounted for the high burden of VDD in the neonates. Furthermore, this study was conducted in a single tertiary level hospital where the patient population may be different from other hospitals. Hence, the results of this study may not necessarily be generalizable to the population.

## 5. Conclusions

VDD was highly prevalent in the neonates studied. Neonates born to HIV-infected mothers were significantly less likely to have VDD compared to neonates born to HIV uninfected mothers. It is recommended to screen for vitamin D status in neonates in the community for generalizability and possibly a randomized clinical trial looking at the benefits of supplementing neonates with VDD in the community.

## Figures and Tables

**Table 1:** Baseline characteristics of the study population.

**Characteristic**	**Frequency ** **(n = 170)**	**Percentage (%)**
**Sex**		
Male	88	51.8
Female	82	48.2
**Gestational Age, weeks**		
<37	62	36.5
37 – 42	106	62.3
> 42	2	1.2
**Birth Weight, grams**		
< 1500	4	2.4
1500 – 2499	38	22.3
≥ 2500	128	75.3
**Breast feeding (BF)**		
BF + Colostrum	85	50.0
BF – Colostrum	8	4.7
No BF	77	45.3

**Table 2:** Factors associated with vitamin d status.

**Factor**	**Vitamin D Status**		**Total (%)**	**p value**
	**Sufficient** ^**∗**^ **(n = 34) (20.0%)**	**Deficient** **(n = 136) (80.0%)**		
**Sex**				
Male	22 (25.0)	66 (75.0)	88	
Female	12 (14.6)	70 (85.4)	82	0.124
**Gestational Age, weeks**				
< 37	14 (22.6)	48 (77.4)	62	
37 – 42	20 (18.5)	88 (81.5)	108	0.554
**Birth Weight, grams**				
< 2500	8 (19.0)	34 (81.0)	42	
≥ 2500	26 (20.3)	102 (79.7)	128	1.000
**Parity**				
Primiparous	11 (17.7)	51 (82.3)	62	
Multiparous	21 (21.2)	78 (78.8)	99	
Grand Multipara	2 (22.2)	7 (77.8)	9	0.808
**Maternal Age, yr**				
< 20	2 (12.5)	14 (87.5)	16	
20 – 29	18 (19.1)	76 (80.9)	94	
≥ 30	14 (23.3)	46 (76.7)	60	0.627
**Maternal HIV**				
Positive	7 (50.0)	7 (50.0)	14	
Negative	27 (17.3)	129 (82.7)	156	0.009
**BF Status**				
With Colostrum	19 (22.4)	66 (77.6)	85	
No Colostrum	0	8 (100.0)	8	
No BF	15 (19.5)	62 (80.5)	77	0.397

^*∗*^Including neonates with normal and insufficient vitamin D levels.

**Table 3:** Maternal HIV status against neonatal vitamin D status.

		**Neonatal Vitamin D status**			
		**Deficient (%)**	**Sufficient (%)**	**Total **	**OR (95% CI), p value**
Overall Study Population					
Maternal HIV status	Positive	7 (50.0)	7 (50.0)	14	0.21
	Negative	129 (82.7)	27 (17.3)	156	(0.06 – 0.77)
	Total	136 (80.0)	34 (20.0)	170	0.009
Among Preterm Neonates					
Maternal HIV status	Positive	4 (66.7)	2 (33.3)	6	0.55
	Negative	44 (78.6)	12 (21.4)	56	(0.06 – 6.79)
	Total	48 (77.4)	14 (22.6)	62	0.61
Among Term Neonates					
Maternal HIV status	Positive	3 (37.5)	5 (62.5)	8	0.11
	Negative	85 (85.0)	15 (15.0)	100	(0.02 – 0.63)
	Total	88 (81.5)	20 (18.5)	108	0.005

**Table 4:** Neonatal vitamin D status against neonatal comorbidity.

		**Neonatal Vitamin D status**		
		**Deficient (%)**	**Sufficient (%)**	**Total **
**Any Comorbidity**	**Present**	77 (81.1)	18 (18.9)	95
	**Absent**	59 (78.7)	16 (21.3)	75
	**Total**	136 (80.0)	34 (20.0)	170

***Odds ratio = 1.16 (95% CI = 0.51 – 2.64, p = 0.699).***

## Data Availability

The data that support the findings of this study are available from the Muhimbili University of Health and Allied Sciences, but restrictions apply to the availability of these data, which were used under license for the current study, and so are not publicly available. Data are however available from the authors upon reasonable request and with permission of Muhimbili University of Health and Allied Sciences.
